# Correlations of apathy with clinical symptoms of Alzheimer’s disease and olfactory dysfunctions: a cross-sectional study

**DOI:** 10.1186/s12883-020-01978-9

**Published:** 2020-11-14

**Authors:** Shu-yang Yu, Teng-hong Lian, Peng Guo, Li-xia Li, Du-yu Ding, Dan-ning Li, Li Liu, Hui Zhao, Yang Hu, Li-jun Zuo, Jun-hua Gao, Qiu-jin Yu, Zhao Jin, Rui-dan Wang, Rong-yan Zhu, Xiao-min Wang, Wei Zhang

**Affiliations:** 1 Department of Neurology, Beijing Tiantan Hospital, Capital Medical University, Beijing, 100070 China; 2 Department of General Internal Medicine, Beijing Tiantan Hospital, Capital Medical University, Beijing, 100070 China; 3 Department of Physiology, Capital Medical University, Beijing, 100069 China; 4 China National Clinical Research Center for Neurological Disease, Beijing Tiantan Hospital, Capital Medical University, Beijing, 100070 China; 5 Center of Parkinson’s Disease, Beijing Institute for Brain Disorders, Beijing, 100053 China; 6 Beijing Key Laboratory on Parkinson Disease, Beijing, 100053 China

**Keywords:** Alzheimer’s disease, Apathy, Olfactory functions, Cognitive functions, Neuropsychiatric symptoms, Activity of daily living

## Abstract

**Background:**

Apathy is one of the most common symptoms of Alzheimer’s disease (AD), however, correlations of apathy with demographic variables, cognitive functions, neuropsychiatric symptoms, activity of daily living and olfactory functions in AD patients are still lacking comprehensive investigations.

**Methods:**

This is a cross-sectional study. Total 124 typical AD patients were consecutively recruited from April 2014 to April 2017. In 124 AD patients, 47 cases (37.9%) were male and 77 cases were female; patients’ age were 43–93 years with an average of 68 years. Patients were divided into AD with apathy (AD-A) and AD with no apathy (AD-NA) groups according to the score of Modified Apathy Evaluation Scale, then were evaluated cognitive functions, neuropsychiatric symptoms and activity of daily living, and tested olfactory functions. Above variables were compared between AD-A and AD-NA groups. Further correlation analyses and linear regression analysis were performed between apathy and above variables.

**Results:**

Compared with AD-NA group, global cognitive level, verbal memory, verbal fluency and activity of daily living were significantly compromised in AD-A group (*P* < 0.002); depression and agitation were severely displayed in AD-A group (*P* < 0.002). Apathy was negatively correlated with global cognitive function, verbal memory, verbal fluency and activity of daily living (*P* < 0.05). There was no significant difference of olfactory functions between the two groups (*P* > 0.002), and correlations between apathy and olfactory threshold, olfactory identification and global olfactory function were significant (*P* < 0.05) but quite weak (|r| < 0.3). Further linear regression analysis showed that only verbal fluency and instrumental activities of daily living were independently associated with apathy.

**Conclusions:**

Independent correlations among apathy, verbal fluency and instrumental activities of daily living in AD patients might be related to the common brain area involved in their pathogeneses.

## Background

Apathy is characterized by the lack of motivation, decreased initiative, akinesia, and emotional indifference. Apathy has been proposed to be a signal of imminent cognitive decline and future risk for dementia in patients with Alzheimer’s disease (AD) [1]. The prevalence of apathy also increases as dementia severity worsens [2]. It is known that the multiple cognitive domains of AD patients are progressively impaired in the process of disease, however, the specific cognitive domain associated with apathy is rarely reported.

Other neuropsychiatric symptoms, such as depression, anxiety and agitation are also common in patients with mild cognitive impairment (MCI) and dementia due to AD. However, the relation between apathy and other neuropsychiatric symptoms in AD, are barely reported and not clear now.

It was reported that patients with MCI and dementia due to AD exhibited significantly worse performance in olfactory identification (ID) compared with healthy individuals [3]. Furthermore, longitudinal studies showed that olfactory identification ID was able to predict cognitive decline due to neuropathology of AD [4]. Olfactory disorder and apathy both could predict the development of AD, however, the relation between them is not clear now, and the relation between apathy and other domains of olfaction, such as olfactory threshold (THR) and discrimination (DIS), are rarely reported.

In a word, this investigation aims to provide comprehensive investigations on correlations between apathy and cognitive functions, neuropsychiatric symptoms, activity of daily living and olfactory functions in AD patients, which may give better understandings of AD with apathy.

## Methods

### Subjects

This is a cross-sectional study. Total 124 AD patients who were community dwelling were consecutively recruited from April 2014 to April 2017. All patients were diagnosed with typical AD phenotype according to the International Working Group-2 criteria [5]. Patients with history of sinus surgery or nasal fracture, depression or other major mental health disorders were excluded.

Patients’ demographic information, including gender, age, disease duration, educational level and smoking condition was collected. Disease duration was based on the retrospective clinical information of the illness timeline. All AD patients were evaluated by Modified Apathy Evaluation Scale (MAES). MAES [6] is an abridged version of an apathy scale designed by Robert Mann [7]. MAES has 14 items, and the score of each item ranges from 0 to 3 (points), representing “not at all”, “slightly”, “some”, and “a lot”, respectively. Thus, the total score of MEAS ranges from 0 to 42 (points), and the higher score indicates more severe apathy. Using a cutoff point of 14 points, the sensitivity and specificity were 66 and 100%, respectively [6]. According to the results of MAES, patients were divided into AD with apathy (AD-A, MAES> 14 points) group and AD with no apathy (AD-NA, MAES ≤14 points) group, respectively.

### Assessments of clinical symptoms

#### Cognitive functions

Patients were evaluated for global cognitive function by Mini-Mental State Examination (MMSE). Score ranges from 0 to 30 (points); lower score indicates more severe cognitive impairment [8]. Individual cognitive domain was assessed by scales as followed:

Memory: Memory was assessed by the Auditory Verbal Learning Test (AVLT) [9]. It includes immediate recall with the score range from 0 to 36 (points), short- delayed recall and long-delayed recall, both with the score range from 0 to 12 (points). Lower score indicates worse memory.

Attention: The Symbol Digit Modalities Test (SDMT) was used to evaluate attention [10] with score range from 0 to 95 (points). Lower score reflects worse attention. The Trail Making Test A (TMT-A) was another scale used for testing attention [11]. Longer time spent in the test (the maximum is 4 min) means worse attention.

Executive function: The Stroop Color-Word Test (SCWT) was used to evaluate executive function [11] with score range from 0 to 50 (points).The lower the score was, the worse the executive function was. The Trail Making Test B (TMT-B) was another scale used for assessing attention [11]. The maximum time used for the test is 4 min. The longer the time consumed, the worse the executive function presented.

Language: The Animal Fluency Test (AFT) was used to assess language [12]. Patients were asked to enumerate animals as more as possible in 1 min. Score was the number of animals enumerated. If the score was lower, the language function was poorer.

Visuospatial ability: The Complex Figure Test (CFT) was used to evaluate visuospatial ability [13] with score range from 0 to 36 (points). Lower score represents worse visuospatial ability.

#### Neuropsychiatric symptoms

Depression was evaluated by the Hamilton Depression Scale (HAMD)-24 items [14] with scores range from 0 to 80 (points), and score > 8 points implies depression. Anxiety was assessed by the Hamilton Anxiety Scale (HAMA)-14 items [15] with score range from 0 to 56 (points) and score > 8 points indicated anxiety. Agitation was tested by the Cohen-Mansfield Agitation Inventory (CMAI) [16] with score range from 0 to 203 (points), and the higher the CMAI score was, the severer the agitation was.

#### Activities of daily living (ADL)

The scales established by Lawton and Brody were used to assess ADL of each patient, including Physical Self-maintenance Scale as basic ADL scale (BADL Scale, score ranges from 6 to 24 points) and Instrumental Activities of Daily Living Scale (IADL Scale, score ranges from 8 to 32 points) [17]. The higher the score was, the worse the ADL was.

#### Test of olfactory functions

Olfactory functions of AD patients were evaluated by Sniffin’ Sticks test, which was from Burghart Messtenik Company, German (product number: LA-13-00005). There were total 112 sticks, among which, 48, 48 and 16 were used for testing olfactory THR, DIS and ID, respectively.

THR: Participants were exposed to n-butyl alcohol from the lowest concentration to the highest one. The score of olfactory THR was the number of Sniffin sticks with the minimal concentration of n-butyl alcohol that participants identified. The lower the score of olfactory THR was, the worse the function of olfactory recognition was.

DIS: Participants were instructed to distinguish the target odor from the other two. The score of olfactory DIS was the number of Sniffin sticks that participants correctly chose. The lower the score of olfactory DIS was, the worse the function of olfactory discrimination was.

ID: Participants were required to identify the odor he (she) smelled among the 4 given answers. The score of olfactory ID was the number of Sniffin sticks that participants correctly answered. The lower the score of olfactory ID was, the worse the function of olfactory identification was.

Overall olfactory function was assessed by summing up the scores of olfactory THR, DIS and ID, which was abbreviated as TDI. Olfactory dysfunction was identified by the criteria provide by a cross-sectional study in 3282 people [18]. If the age of an individual was between 36 and 55 years old, and male one with TDI score ≤ 24 points, or female one with TDI score ≤ 28 points, was thought to have olfactory dysfunction; if the age of an individual was > 55 years old, a female or male one with TDI score ≤ 19 points was thought to have olfactory dysfunction.

### Data analyses

Statistical analyses were performed with SPSS Statistics 20.0 (IBM Corporation, New York, USA). Continuous variables were presented as mean ± standard deviations and compared by 2-tailed t-test if they were normally distributed, and presented as median (quartile) and compared by nonparametric test if they were not normally distributed. Discrete variables were compared by Chi square test. Demographic information, cognitive functions, neuropsychiatric symptoms, olfactory functions and activity of daily life were compared between AD-A and AD-NA groups. Because there were total 25 comparisons between the two groups,Bonferroni correction was performed and the corrected *P* value was significant when it was less than 0.002(0.05/25 = 0.002). Due to MEAS scores were ordinal data, Spearman correlation analyses were performed between MAES scores and related factors. Further multiple linear regression analysis was performed between apathy and variables that had linear correlations with MAES score. Correlations were considered as significant when *P* < 0.05 and |r| > 0.3.

## Results

In this study, among 124 AD patients, 47 cases (37.9%) were male, and age from 45 to 93 years with an average of 68 years**;** 77 cases were female and age was 43–88 years with an average of 68 years. Sixty out of 124 (48.4%) AD patients had apathy. The mean apathy score of AD-A group was significantly higher than that of AD-NA group [26.0(18.3 ~ 35.6) vs. 4.5(0.3 ~ 9.0), *P* < 0.002].

Demographic information, cognitive functions, neuropsychiatric symptoms, ADL and olfactory functions were compared between AD-A and AD-NA groups. The results showed that, gender, age, disease duration, educational level and smoking condition were not significantly different between two groups (*P* > 0.002) (Table 1).

**Table 1:** Demographic variables of AD-A^a^ and AD-NA^b^ groups

	AD-A group (60 cases)	AD-NA group (64 cases)	***P*** value
Gender			0.580
Male [case (%)]	21(35.0%)	26(40.6%)	
Female [case (%)]	39(65.0%)	38(59.4%)	
Age [year, mean ± SD]	67.6 ± 9.9	68.4 ± 10.6	0.663
Disease duration [year, median (quartile)]	3.0(2.0 ~ 5.0)	2.0(1.6 ~ 4.0)	0.225
Educational level			0.238
Illiteracy [case (%)]	4(6.7%)	2(3.1%)	
Primary school [case (%)]	12(20.0%)	8(12.5%)	
Middle school [case (%)]	13(21.7%)	17(26.6%)	
High school [case (%)]	20(33.3%)	16(25.0%)	
College and above [case (%)]	11(18.3%)	21(32.8%)	
Smoking condition			0.364
Smoking [case (%)]	14(23.3%)	10(15.6%)	
Non-smoking [case (%)]	46(76.7%)	54(84.4%)	

*P < 0.002 after Bonferroni correction.^a^*AD-A* Alzheimer disease with apathy, ^b^*AD-NA* Alzheimer disease with no apathy

AD-A group had more serious cognitive dysfunctions, including global cognitive function, immediate recall, short-delayed recall, verbal memory and verbal fluency than AD-NA group (*P* < 0.002). Depression and agitation were more serious in AD-A group than AD-NA group (*P* < 0.002). AD-A group also had worse ADL, including BADL and IADL (*P* < 0.002) (Table 2). Further correlation analyses indicated that apathy was negatively correlated with global cognitive function, immediate recall, short-delayed recall, verbal memory and verbal fluency, and positively correlated with ADL, including BADL and IADL (*P* < 0.05) (Table 3). There was no significant difference of olfactory functions between two groups (*P* > 0.05) (Table 4). Correlation analyses implied that apathy was negatively correlated with olfactory THR, ID and TDI (*P* < 0.05). However, the absolute value of coefficients was less than 0.3 (Table 5). Further linear regression analysis was made between apathy and variables that had linear correlations with it, including MMSE, AVLT, AFT, BADL and IADL (Table 6). The results showed that only the scores of AFT and IADL were independently associated with MAES score (*P* < 0.05).

**Table 2:** Clinical Symptoms of AD-A^a^ and AD-NA^b^ groups

	AD-A group (60 cases)	AD-NA group (64 cases)	***P*** value
**Global cognitive function**			
MMSE^c^ [points, median (quartile)]	18.0(10.0 ~ 25.0)	26.0(21.3 ~ 29.0)	0.000*
**Memory**			
AVLT^d−^Immediate recall [points, median (quartile)]	9.0(6.0 ~ 12.0)	13.0(8.8 ~ 17.0)	0.001*
AVLT-Short-delayed recall [points, median (quartile)]	0(0 ~ 3.0)	2.5(0 ~ 6.5)	0.000*
AVLT-long-delayed recall [points, median (quartile)]	0(0 ~ 3.0)	3.0(0 ~ 6.0)	0.003
**Attention**			
SDMT^e^ [points, median (quartile)]	22.0(12.0 ~ 29.0)	21.0(15.0 ~ 38.0)	0.445
TMT^f^-A-time consuming [seconds, median (quartile)]	108.0(67.0 ~ 187.5)	75.0(60.0 ~ 122.0)	0.075
**Executive function**			
SCWT^g^ [points, median (quartile)]	46.5(41.5 ~ 49.25)	48.0(42.3 ~ 50.0)	0.265
TMT-B- time consuming [seconds, median (quartile)]	238.0(194.0 ~ 240.0)	222.0(168.5 ~ 240.0)	0.550
**Language**			
AFT^h^ (points, mean ± SD)	11.5(8.3 ~ 14.8)	15.5(12.0 ~ 20.0)	0.000*
**Visuospatial function**			
CFT^i^-Copy [points, median (quartile)]	22.0(5.0 ~ 32.0)	26.0(10.5 ~ 34.0)	0.427
**Neuropsychiatric symptoms**			
HAMD^j^ [points, median (quartile)]	10.0(3.0 ~ 15.0)	4.0(1.0 ~ 8.0)	0.001*
HAMA^k^ [points, median (quartile)]	6.0(3.0 ~ 12.0)	3.0(0 ~ 6.0)	0.004
CMAI^l^ [points, median (quartile)]	29.0(29.0 ~ 38.0)	29.0(29.0 ~ 29.0)	0.000*
**Activities of daily living**			
BADL^m^ [points, median (quartile)]	6.0(6.0 ~ 10.0)	6.0(6.0 ~ 6.0)	0.001*
IADL^o^ [points, median (quartile)]	11.5(8.0 ~ 25.5)	8.0(8.0 ~ 11.0)	0.000*

*P < 0.002 after Bonferroni correction, ^a^*AD-A* Alzheimer disease with apathy, ^b^*AD-NA* Alzheimer disease with no apathy, ^c^ Mini-Mental State Examination, ^d^*AVLT* Auditory Verbal Learning Test, ^e^*SDMT* Symbol Digit Modalities Test, ^f^*TMT* Trial Making Test, ^g^*SCWT* Stroop Color Word Test, ^h^*AFT* Animal Fluency Test, ^i^*CFT* Complex Figure Test, ^j^*HAMD* Hamilton Depression Scale, ^k^*HAMA* Hamilton Anxiety Scale, ^l^*CMAI* Cohen- Mansfield Agitation Inventory, ^m^Basic Activity of Daily Living, ^o^Instrucmental Activity of Daily Living

**Table 3:** Spearman correlation analyses between apathy and related factors in Alzheimer disease patients

Factors	Coefficient	***P*** value
Age [year, mean ± SD]	−0.057	0.530
Disease duration [year, median (quartile)]	0.113	0.220
**Global cognitive function**		
MMSE^a^ [points, median (quartile)]	− 0.516	0.000*
**Memory**		
AVLT^b−^ Immediate recall [points, median (quartile)]	−0.346	0.000*
AVLT- Short-delayed recall [points, median (quartile)]	−0.328	0.001*
AVLT- Long-delayed recall [points, median (quartile)]	−0.270	0.005*
**Attention**		
SDMT^c^ [points, median (quartile)]	−0.185	0.184
TMT^d^-A-time consuming [seconds, median (quartile)]	−0.042	0.752
**Executive function/attention**		
SCWT^e^- correct number [points, median (quartile)]	−0.126	0.354
TMT-B- time consuming [seconds, median (quartile)]	−0.123	0.361
**Language**		
AFT^f^ (points, mean ± SD)	−0.453	0.000*
**Visuospatial function**		
CFT^g^-Copy [points, median (quartile)]	−0.176	0.129
**Neuropsychiatric symptoms**		
HAMD^h^ [points, median (quartile)]	0.115	0.365
HAMA^i^ [points, median (quartile)]	0.128	0.314
CMAI^j^ [points, median (quartile)]	−0.096	0.450
**Activities of daily living**		
BADL^k^ [points, median (quartile)]	0.415	0.000*
IADL^l^ [points, median (quartile)]	0.444	0.000*

*P < 0.05, ^a^Mini-Mental State Examination, ^b^*AVLT* Auditory Verbal Learning Test, ^c^*SDMT* Symbol Digit Modalities Test, ^d^*TMT* Trial Making Test, ^e^*SCWT* Stroop Color Word Test, ^f^*AFT* Animal Fluency Test, ^g^*CFT* Complex Figure Test, ^h^*HAMD* Hamilton Depression Scale, ^i^*HAMA* Hamilton Anxiety Scale, ^j^*CMAI* Cohen- Mansfield Agitation Inventory, ^k^Basic Activity of Daily Living, ^l^Instrucmental Activity of Daily Living

**Table 4:** Olfactory functions of AD-A^a^ and AD-NA^b^ groups

	AD-A group (60 cases)	AD-NA group (64 cases)	***P*** value
THR^c^ [points, median (quartile)]	4.0(2.0 ~ 6.0)	5.0(3.0 ~ 6.0)	0.274
DIS^d^ [points, median (quartile)]	8.0(4.5 ~ 10.0)	8.0(6.0 ~ 11.0)	0.081
ID^e^ [points, median (quartile)]	8.0(5.0 ~ 10.5)	9.0(7.0 ~ 12.3)	0.068
TDI^f^[scores, median (quartile)]	21.0(14.0 ~ 25.0)	22.0(17.8 ~ 29.0)	0.074

*P < 0.002 after Bonferroni correction, ^a^*AD-A* Alzheimer disease with apathy, ^b^*AD-NA* Alzheimer disease with no apathy, ^c^Olfactory Threshold, ^d^Olfactory Discrimination, ^e^Olfactory Identification, ^f^Sum of olfactory threshold, discrimination and identification

**Table 5:** Spearman correlation analyses between apathy and olfactory symptoms in Alzheimer disease patients

	Coefficient	***P*** value
THR^a^ [points, median (quartile)]	−0.254	0.012*
DIS^b^ [points, median (quartile)]	−0.154	0.127
ID^c^ [points, median (quartile)]	−0.235	0.019*
TDI^d^ [scores, median (quartile)]	−0.242	0.016*

*P < 0.05, ^a^Olfactory Threshold, ^b^Olfactory Discrimination, ^c^Olfactory Identification, ^d^Sum of olfactory threshold, discrimination and identification

**Table 6:** Multiple linear regression analyses between apathy and related factors

Factor	Included variables	Excluded variables	***P*** value	∆R^**2**^	Adjusted R^**2**^
**MAES**^**a**^	AFT^b^ IADL^d^	MMSE^c^ AVLT^e−^ Immediate recall AVLT- Short-delayed recall AVLT- Long-delayed recall BADL^f^	0.000*	0.230	0.215

*P < 0.05, ^a^Modified Apathy Evaluation Scale, ^b^AFT: Animal Fluency Test, ^c^Mini-Mental State Examination, ^d^Instrumental Activity of Daily Living, ^e^AVLT: Auditory Verbal Learning Test, ^f^Basic Activity of Daily Living

## Discussion

In this investigation, some of participants were with MCI due to AD, which also had apathy. Patients with MCI due to AD and AD dementia had the same pathological changes of AD, therefore, we did not specifically distinguish MCI due to AD from AD dementia. We hope to figure out the common influencing factors of apathy in AD patients. In AD patients recruited in this study, the frequency of apathy reached up to 48.4%. Thus, clinician should pay great attention to this easily ignored symptom of AD patients.

The relation between apathy and global cognitive function was investigated in this research. Global cognitive function was significantly impaired in AD-A group than AD-NA group (*P* < 0.002). The relation between apathy and individual cognitive domains was then explored. Memory was the first one we investigated. The results showed that scores of immediate recall and short-delayed recall were significantly lower in AD-A group than AD-NA group (*P* < 0.002), and both were negatively correlated with the scores of MAES (*P* < 0.05). It was reported that one pivotal pathology of apathy in AD was the lack of cholinergic transmitters in the frontal and marginal lobes, especially in the frontal lobe [19]. Interim results from another study suggested that combination of donepezil, an acetylcholinesterase inhibitor, and choline alphoscerate, a cholinergic precursor, alleviated apathy and reduced caregiver distress [20]. Open label studies with cholinesterase inhibitors, including donepezil, rivastigmine and galantamine, showed an improvement in apathy for AD patients [21]. Therefore, the damage of cholinergic function might connect apathy and memory decline in AD patients.

In this research, AFT score was significantly lower in AD-A group than AD-NA group (*P* < 0.002), and also negatively correlated with MAES score (*P* < 0.05). AFT is widely used for assessing semantic function of language, which mainly reflects the function of temporal lobe. The database from the Alzheimer Disease Neuroimaging Initiative, a larger study, showed that cortical thinning in temporal cortex was associated with more severe apathy over time after correcting for multiple covariates, such as sex, age, APOE genotype, premorbid intelligence, memory performance, processing speed, antidepressant use, and AD duration [22]. There are extensive projections from temporal lobe to frontal lobe, so the damage in temporal lobe may lead to apathy and problem of verbal fluency at the same time in AD patients.

In this study, AD-A group had more serious depression than AD-NA group (*P* < 0.002). This might because that apathy and depression are often comorbid. Apathy and depression have similar clinical manifestations, and share some common pathological changes, such as hypoperfusion. However, they also have their unique pathogenic processes [23], which might explain that no significant correlation was found between depression and apathy in this study (*P* > 0.05). Apathy was specifically correlated with hypometabolism in left orbitofrontal areas while depression was associated with hypometabolism in left dorsolateral prefrontal regions [24]. Apathy score was positively correlated with PIB signal in bilateral frontal and right anterior cingulate cortices, but no correlation was found between PIB and depression [25]. It was reported that apathy but not depression was strongly associated with the transition from MCI to AD [26].

In this study, agitation was more serious in AD-A group than AD-NA group (*P* < 0.002). Apathy and agitation in AD patients may share common mechanisms. For example, it was reported that the scores of apathy and agitation were both correlated with the elevated level of Aβ1–42 [27] and the decreased volume of anterior cingulate [28]. Therefore, it can be speculated that both apathy and agitation may simultaneously worsened when the common mechanisms they share become serious. However, they also have their own pathogeneses. For example, it was found that apathy was positively correlated to hypofrontality [24] as well as increased gamma-aminobutyric acid level in serum [29], and agitation was connected with the damage of locus coeruleus [30] and positively related to increased brain derived neurotrophic factor level in plasma [31] . The specific pathogeneses may explain why agitation was not significantly correlated with apathy in this study (|r| < 0.3).

We found that apathy was associated with both basic and instrumental ADL in AD patients (*P* < 0.002). There might be a few potential explanations. First, the presence of apathy was more common in dementia patients with worse ADL; second, patients with reduced ADL were more likely to have aggravated apathy.

In this study, the scores of olfactory THR, DIS, ID and TDI were not significantly different between AD-A and AD-NA groups (*P* > 0.002), and further correlation analyses showed that MEAS score was positively correlated with the scores of olfactory THR, ID and TDI (*P* < 0.05). However, the absolute value of coefficient was < 0.3, which meant correlations between apathy and olfactory dysfunctions was quite weak. These contradictory results might be due to limited sample number of 124 patients in this study, which was more likely to cause the result to be false positive. Therefore, further study with larger sample size is much needed. However, other study has reported there was a correlation between apathy and olfactory dysfunctions. An exploration comparing 172 patients with AD dementia, 112 patients with MCI due to AD and 132 controls by using olfactory ID performance showed that the two types of patients were significantly worse in identifying odors, and smell identification deficit was significantly correlated with the degree of apathy, but not depression or other neuropsychiatric symptoms. Importantly, this correlation was observed even after controlling for the severity of dementia [32]. The anatomic proximity of the olfactory network and apathy-related structures might provide a potential explanation for the close correlation between olfaction and apathy [4].

In this research, a linear regression analysis was made between MAES score and variables that had linear correlations with apathy, including MMSE, AVLT, AFT, BADL and IADL. The results showed that only the scores of AFT and IADL were independently associated with MAES score (*P* < 0.05). AFT is widely used to test verbal fluency, which reflects semantic function as well as executive function. IADL involves ability of planning and executive function. Executive dysfunction is closely connected with hypofrontality, which also participates in the pathogenesis of apathy. Therefore, we speculate that hypofrontality may be the connection of apathy, verbal fluency and IADL.

The strength of this research is total 11 scales and Sniffin’ Sticks test were used to comprehensively evaluate the clinical symptoms of AD. This investigation also has deficiencies. The sample size was relatively insufficient, and healthy people as control were not included. We did not consider contextual factors that could impact on motivation, such as whether patients were provided with activities that might interest them, and whether the patients’ background was enriched to avoid social isolation. These deficiencies are needed to be avoided in the future study.

## Conclusions

In summary, apathy is a very common symptom of AD. It is independently correlated with the impairments of verbal fluency and IADL, which may reflect the association of brain area involved in their pathogeneses.

## Data Availability

The datasets used and/or analyzed during the current study are available from the corresponding author on reasonable request.

## Abbreviations

AD: Alzheimer disease
AD-A: Alzheimer disease with apathy
AD-NA: Alzheimer disease with no apathy
MCI: mild cognitive impairment
MAES: Modified Apathy Evaluation Scale
MMSE: Mini-Mental State Examination
AVLT: Auditory Verbal Learning Test
SDMT: Symbol Digit Modalities Test
TMT: Trail Making Test
SCWT: Stroop Color-Word Test
AFT: Animal Fluency Test
CFT: Complex Figure Test
HAMD: Hamilton Depression Scale
HAMA: Hamilton Anxiety Scale
CMAI: Cohen-Mansfield Agitation Inventory
BADL: Basic Activities of Daily Living
IADL: Instrumental Activities of Daily Living
THR: Olfactory Threshold
DIS: Olfactory Discrimination
ID: Olfactory Identification
TDI: Sum of Olfactory Threshold, Discrimination and Identification
